# Supplementary material for: Bayesian meta-analysis reveals the mechanistic role of slow oscillation-spindle coupling in sleep-dependent memory consolidation
Source: eLife. 2025 Oct 8;13:RP101992. doi: 10.7554/eLife.101992 (PMC12507438; doi:10.7554/eLife.101992)
Supplement: Supplementary file 2. — Example plots demonstrating the diagnostic process for each fitted Bayesian model, including posterior predictive checks, trace plots, and autocorrelation plots. [file elife-101992-supp2.pdf]

Supplementary File 2: Model Diagnostics

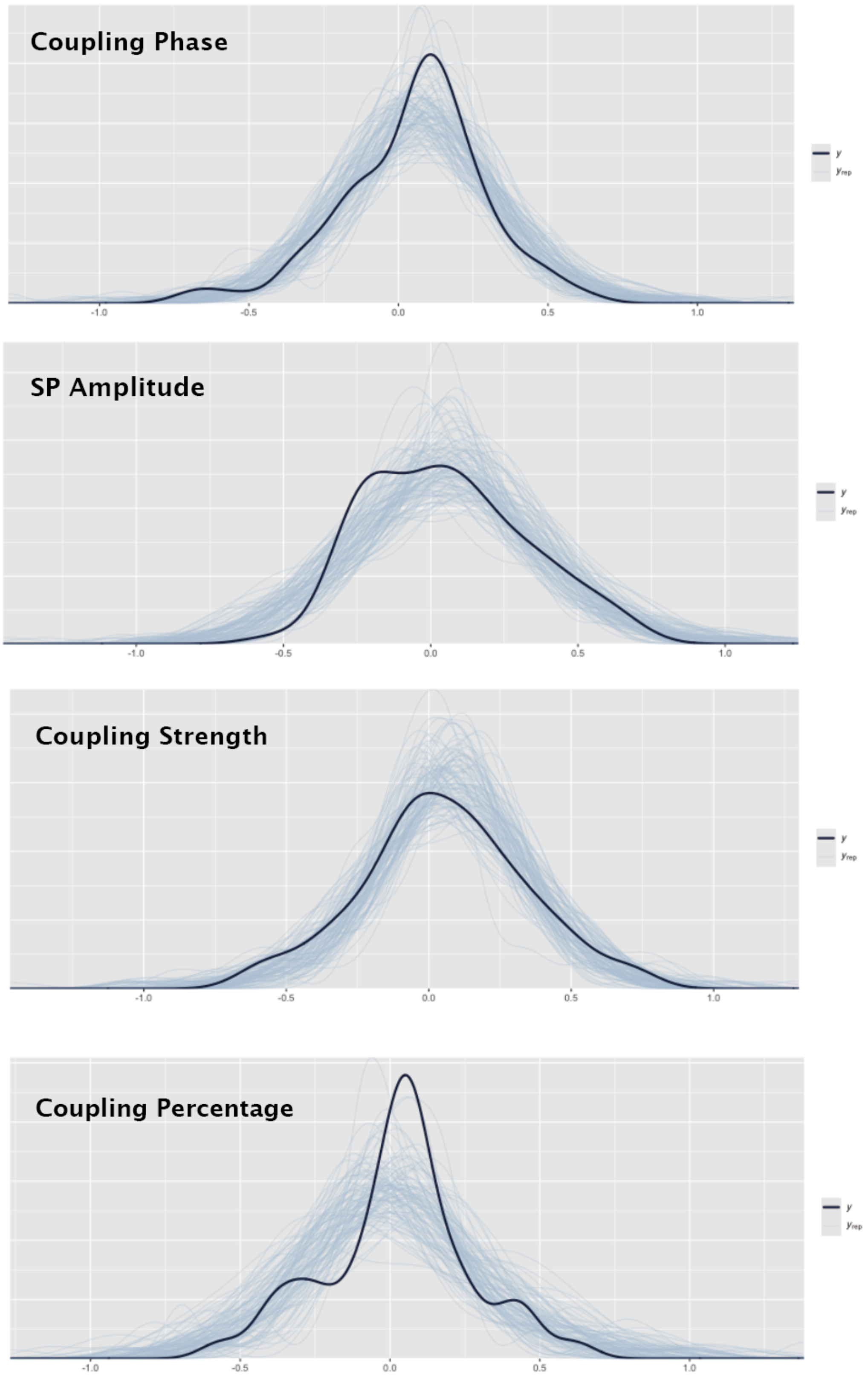

Figure 1: Posterior predictive check of overall models of coupling-memory association measures

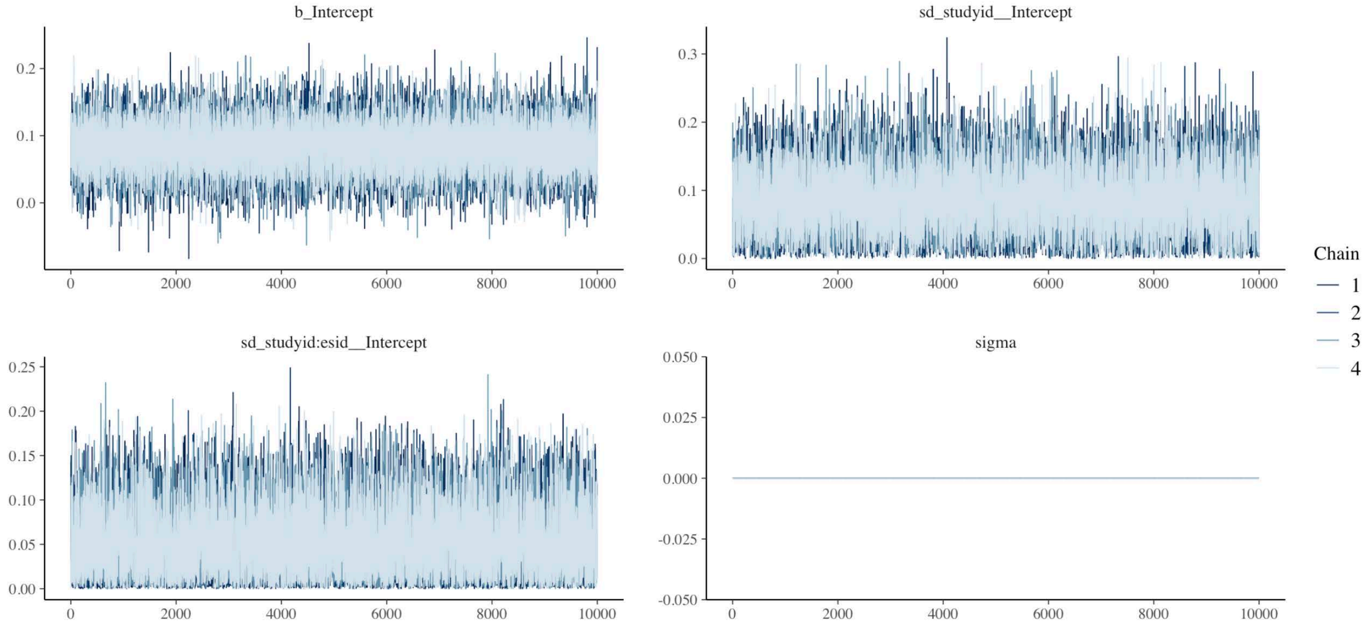

Figure 2: Example of the trace plot used to assess the convergence of models. Well-mixed chains showing no trends or drifts over iterations indicate successful convergence.

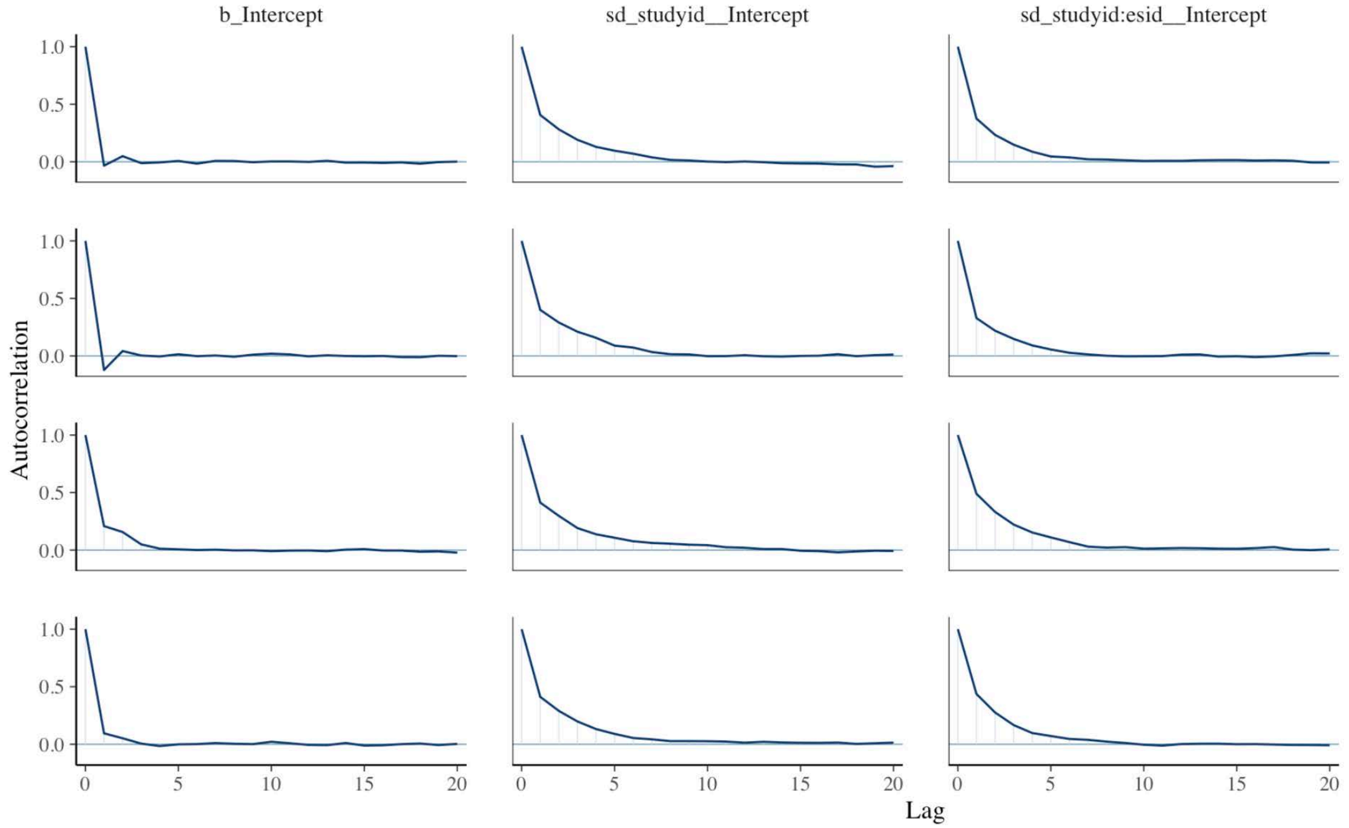

Figure 3: Example of the autocorrelation plot used to assess the independence of samples across iterations, with a rapid decay of autocorrelation indicating efficient sampling and low within-chain redundancy.
