# Supplementary material for: Bayesian meta-analysis reveals the mechanistic role of slow oscillation-spindle coupling in sleep-dependent memory consolidation
Source: eLife. 2025 Oct 8;13:RP101992. doi: 10.7554/eLife.101992 (PMC12507438; doi:10.7554/eLife.101992)
Supplement: Supplementary file 3. [file elife-101992-supp3.pdf]

Supplementary File 3: Effect size-level forest plot

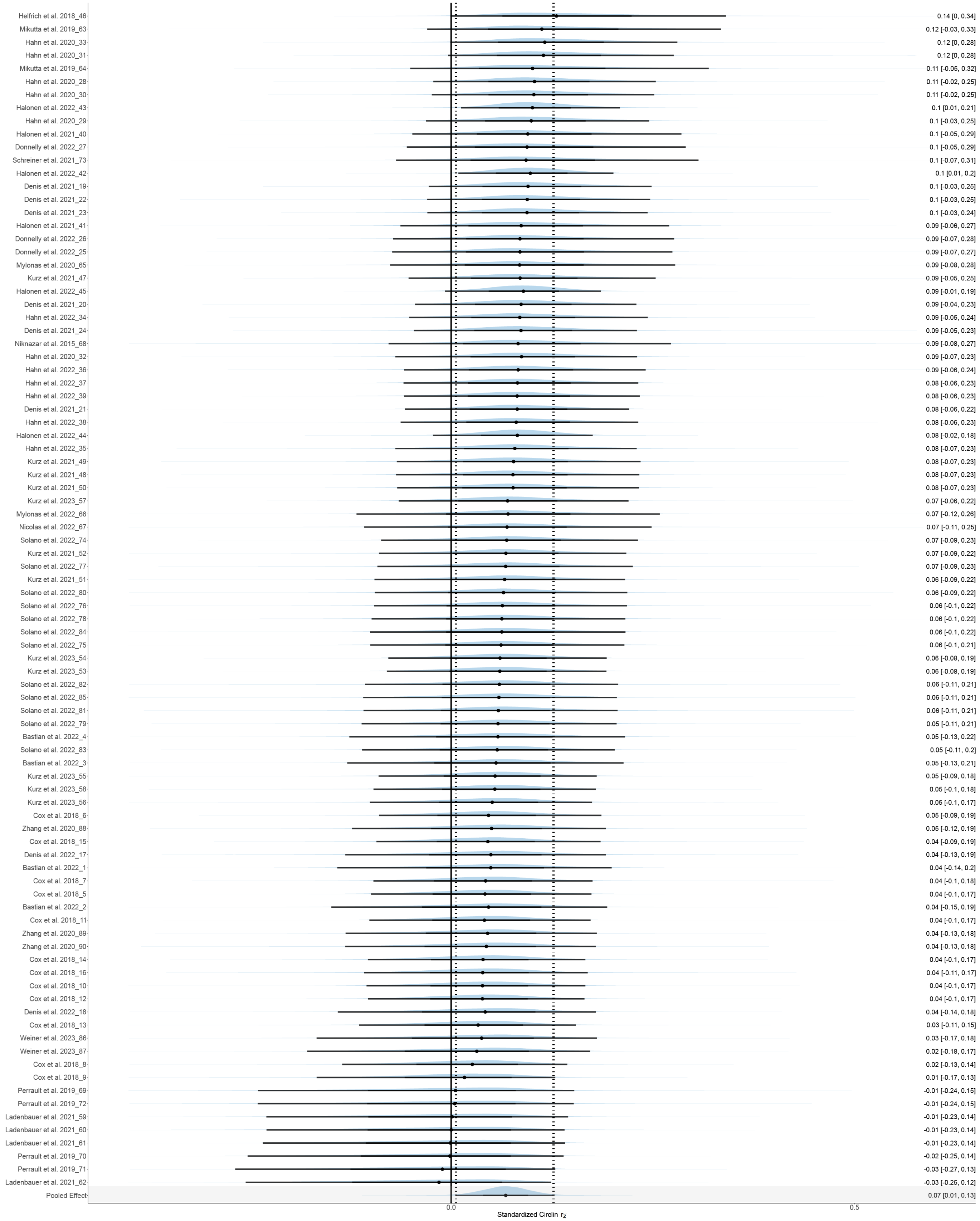

Figure 1: Effect size-level forest plot for the overall model of coupling phase

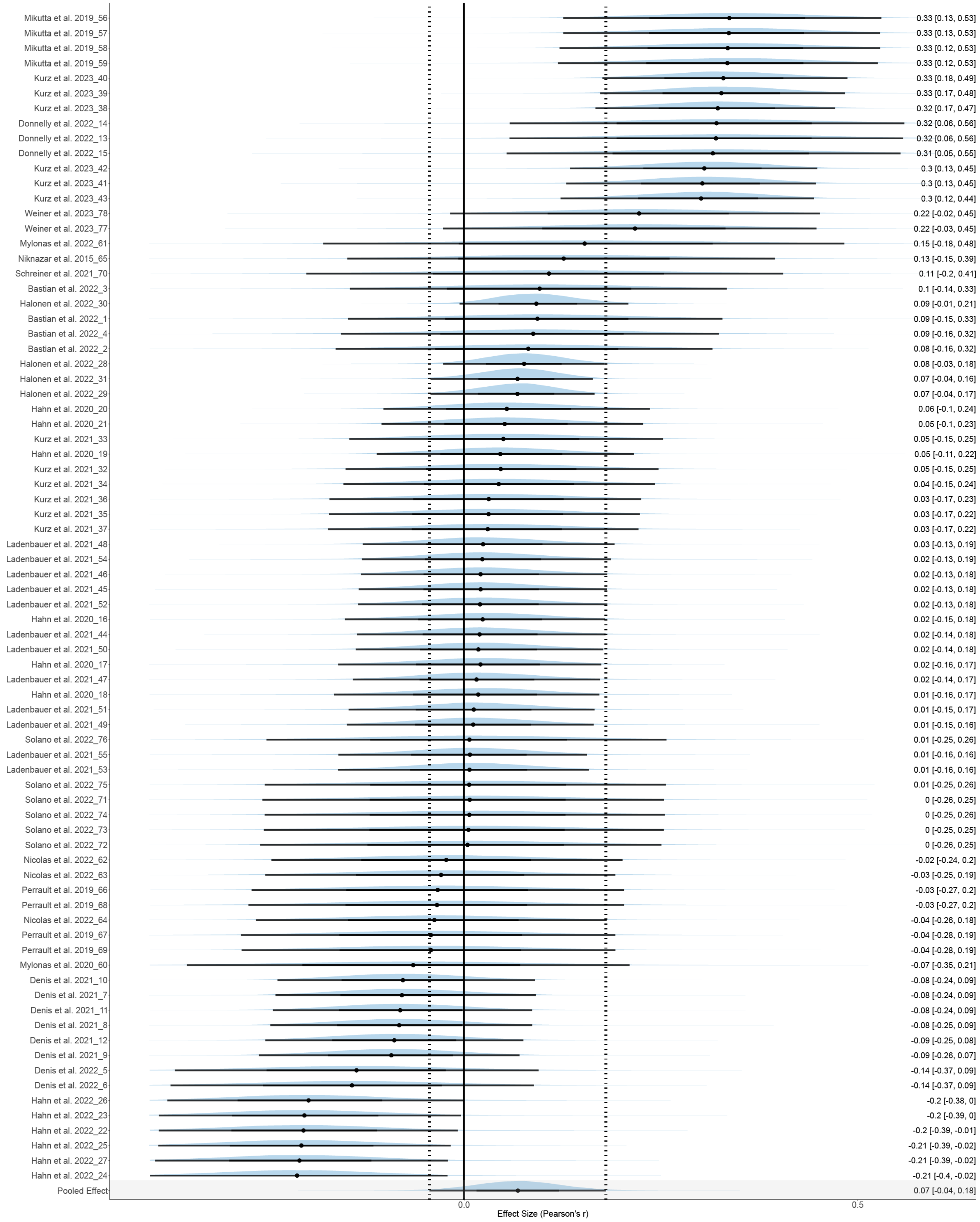

Figure 2: Effect size-level forest plot for the overall model of spindle amplitude

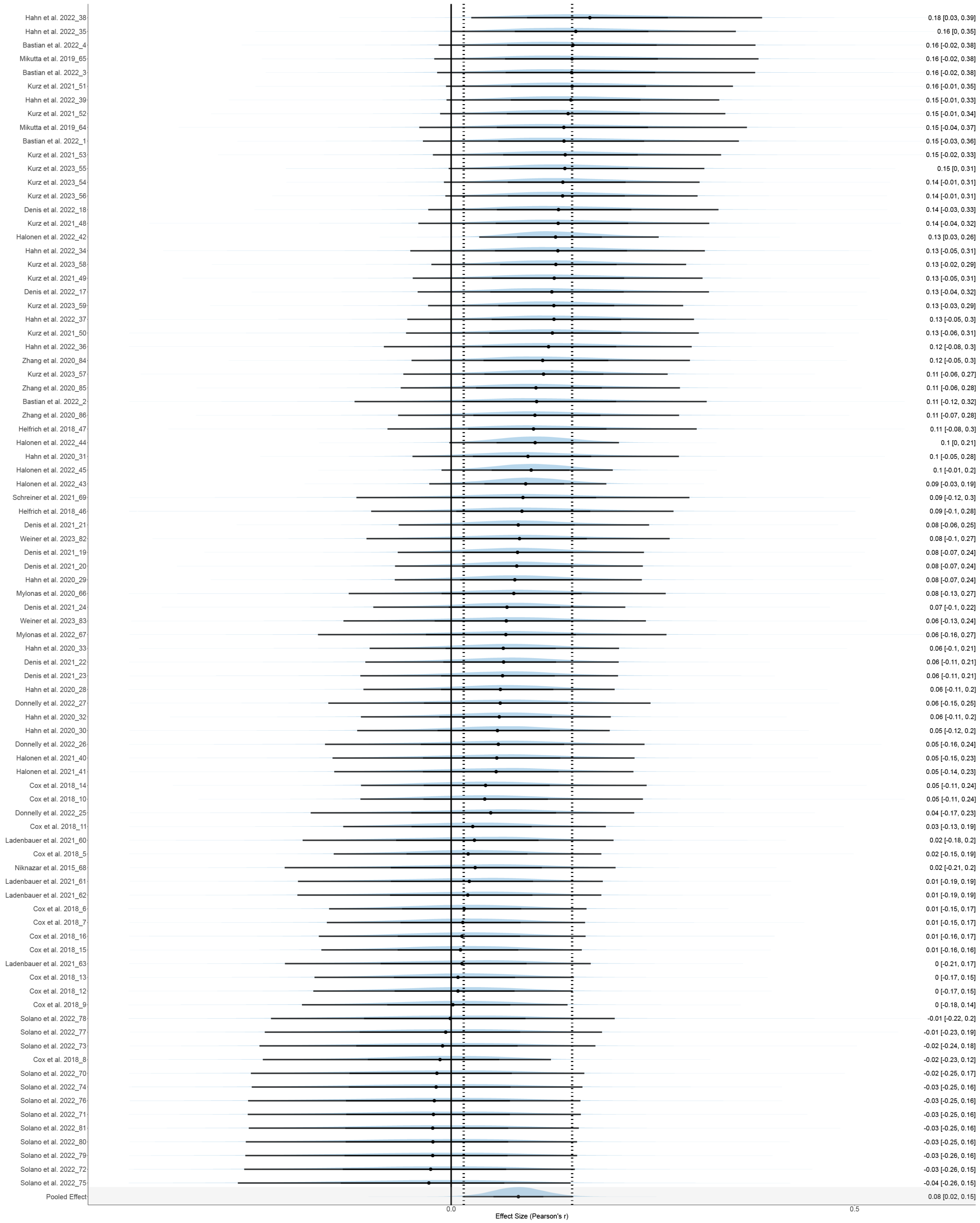

Figure 3: Effect size-level forest plot for the overall model of coupling strength

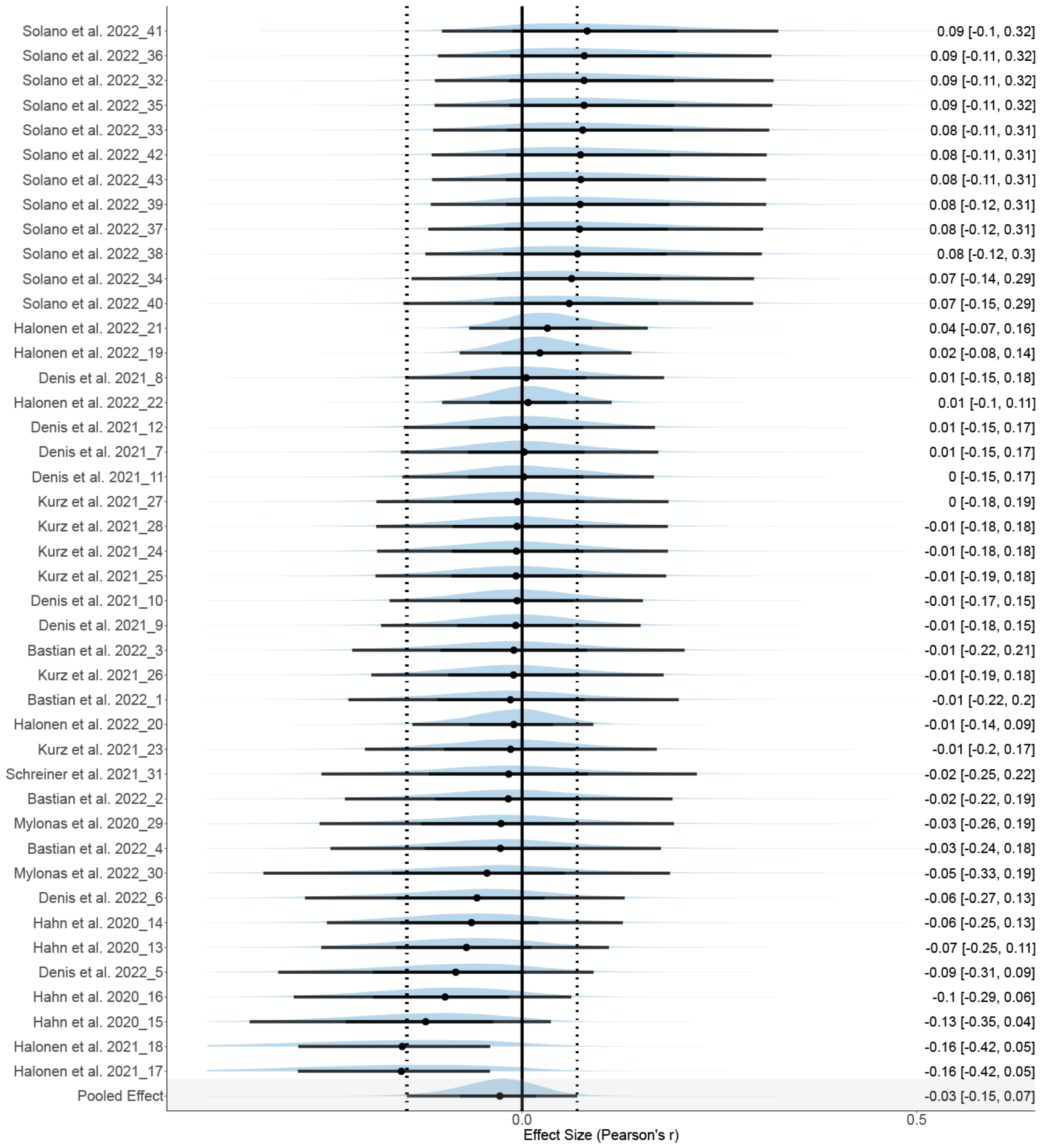

Figure 4: Effect size-level forest plot for the overall model of coupling percentage

Notes. Overall model forest plot at the effect size-level. The solid vertical line represents the mean correlation coefficient under the null hypothesis. Dashed lines indicate the 95% credible interval (*CrI*) of the pooled effect size. The black point and error bar for each effect size shows the adjusted estimation of effect size and 95% *CrI* combining data and prior information, with corresponding values reported in the right side of the figure. On the left, numbers reported after publication years correspond to the esid column labeled in Source data of Figure 4, 6-8.
