# Supplementary material for: Bayesian meta-analysis reveals the mechanistic role of slow oscillation-spindle coupling in sleep-dependent memory consolidation
Source: eLife. 2025 Oct 8;13:RP101992. doi: 10.7554/eLife.101992 (PMC12507438; doi:10.7554/eLife.101992)
Supplement: Supplementary file 4. [file elife-101992-supp4.pdf]

Supplementary File 4: Pareto *K* diagnosis statistics

Pareto k diagnosis statistics for the phase model								
ESID	Study	Pareto k	ESID	Study	Pareto k	ESID	Study	Pareto k
1	Bastian et al. 2022	0.204	31	Hahn et al. 2020	0.401	61	Ladenbauer et al. 2021	0.324
2	Bastian et al. 2022	0.221	32	Hahn et al. 2020	0.455	62	Ladenbauer et al. 2021	0.358
3	Bastian et al. 2022	0.282	33	Hahn et al. 2020	0.571	63	Mikutta et al. 2019	0.397
4	Bastian et al. 2022	0.247	34	Hahn et al. 2022	0.273	64	Mikutta et al. 2019	0.229
5	Cox et al. 2018	0.339	35	Hahn et al. 2022	0.185	65	Mylonas et al. 2020	0.399
6	Cox et al. 2018	0.401	36	Hahn et al. 2022	0.376	66	Mylonas et al. 2022	0.395
7	Cox et al. 2018	0.304	37	Hahn et al. 2022	0.332	67	Nicolas et al. 2022	0.434
8	Cox et al. 2018	0.259	38	Hahn et al. 2022	0.367	68	Niknazar et al. 2015	0.489
9	Cox et al. 2018	0.255	39	Hahn et al. 2022	0.222	69	Perrault et al. 2019	0.320
10	Cox et al. 2018	0.296	40	Halonen et al. 2021	0.453	70	Perrault et al. 2019	0.237
11	Cox et al. 2018	0.357	41	Halonen et al. 2021	0.417	71	Perrault et al. 2019	0.345
12	Cox et al. 2018	0.279	42	Halonen et al. 2022	0.705 *	72	Perrault et al. 2019	0.210
13	Cox et al. 2018	0.377	43	Halonen et al. 2022	0.498	73	Schreiner et al. 2021	0.391
14	Cox et al. 2018	0.421	44	Halonen et al. 2022	0.52	74	Solano et al. 2022	0.103
15	Cox et al. 2018	0.356	45	Halonen et al. 2022	0.551	75	Solano et al. 2022	0.166
16	Cox et al. 2018	0.251	46	Helfrich et al. 2018	0.531	76	Solano et al. 2022	0.195
17	Denis et al. 2022	0.432	47	Kurz et al. 2021	0.365	77	Solano et al. 2022	0.125
18	Denis et al. 2022	0.403	48	Kurz et al. 2021	0.255	78	Solano et al. 2022	0.130
19	Denis et al. 2021	0.331	49	Kurz et al. 2021	0.328	79	Solano et al. 2022	0.102
20	Denis et al. 2021	0.324	50	Kurz et al. 2021	0.322	80	Solano et al. 2022	0.187
21	Denis et al. 2021	0.373	51	Kurz et al. 2021	0.285	81	Solano et al. 2022	0.187
22	Denis et al. 2021	0.380	52	Kurz et al. 2021	0.369	82	Solano et al. 2022	0.141
23	Denis et al. 2021	0.235	53	Kurz et al. 2023	0.289	83	Solano et al. 2022	0.167
24	Denis et al. 2021	0.403	54	Kurz et al. 2023	0.304	84	Solano et al. 2022	0.291
25	Donnelly et al. 2022	0.253	55	Kurz et al. 2023	0.304	85	Solano et al. 2022	0.197
26	Donnelly et al. 2022	0.288	56	Kurz et al. 2023	0.413	86	Weiner et al. 2023	0.340
27	Donnelly et al. 2022	0.278	57	Kurz et al. 2023	0.324	87	Weiner et al. 2023	0.347
28	Hahn et al. 2020	0.305	58	Kurz et al. 2023	0.224	88	Zhang et al. 2020	0.281
29	Hahn et al. 2020	0.322	59	Ladenbauer et al. 2021	0.272	89	Zhang et al. 2020	0.342
30	Hahn et al. 2020	0.293	60	Ladenbauer et al. 2021	0.288	90	Zhang et al. 2020	0.395

Figure 1: Pareto *k* diagnostic statistics for the coupling phase model

Pareto k diagnosis statistics for the SP amplitude model								
ESID	Study	Pareto k	ESID	Study	Pareto k	ESID	Study	Pareto k
1	Bastian et al. 2022	0.224	27	Hahn et al. 2022	0.303	53	Ladenbauer et al. 2021	0.357
2	Bastian et al. 2022	0.173	28	Halonen et al. 2022	0.658	54	Ladenbauer et al. 2021	0.283
3	Bastian et al. 2022	0.291	29	Halonen et al. 2022	0.591	55	Ladenbauer et al. 2021	0.407
4	Bastian et al. 2022	0.195	30	Halonen et al. 2022	0.529	56	Mikutta et al. 2019	0.292
5	Denis et al. 2022	0.453	31	Halonen et al. 2022	0.737 *	57	Mikutta et al. 2019	0.313
6	Denis et al. 2022	0.489	32	Kurz et al. 2021	0.354	58	Mikutta et al. 2019	0.321
7	Denis et al. 2021	0.411	33	Kurz et al. 2021	0.302	59	Mikutta et al. 2019	0.270
8	Denis et al. 2021	0.389	34	Kurz et al. 2021	0.247	60	Mylonas et al. 2020	0.581
9	Denis et al. 2021	0.324	35	Kurz et al. 2021	0.345	61	Mylonas et al. 2022	0.576
10	Denis et al. 2021	0.342	36	Kurz et al. 2021	0.342	62	Nicolas et al. 2022	0.401
11	Denis et al. 2021	0.427	37	Kurz et al. 2021	0.252	63	Nicolas et al. 2022	0.347
12	Denis et al. 2021	0.371	38	Kurz et al. 2023	0.311	64	Nicolas et al. 2022	0.393
13	Donnelly et al. 2022	0.413	39	Kurz et al. 2023	0.286	65	Niknazar et al. 2015	0.568
14	Donnelly et al. 2022	0.381	40	Kurz et al. 2023	0.426	66	Perrault et al. 2019	0.309
15	Donnelly et al. 2022	0.336	41	Kurz et al. 2023	0.485	67	Perrault et al. 2019	0.218
16	Hahn et al. 2020	0.500	42	Kurz et al. 2023	0.339	68	Perrault et al. 2019	0.265
17	Hahn et al. 2020	0.403	43	Kurz et al. 2023	0.353	69	Perrault et al. 2019	0.300
18	Hahn et al. 2020	0.428	44	Ladenbauer et al. 2021	0.298	70	Schreiner et al. 2021	0.542
19	Hahn et al. 2020	0.442	45	Ladenbauer et al. 2021	0.352	71	Solano et al. 2022	0.120
20	Hahn et al. 2020	0.458	46	Ladenbauer et al. 2021	0.334	72	Solano et al. 2022	0.288
21	Hahn et al. 2020	0.442	47	Ladenbauer et al. 2021	0.281	73	Solano et al. 2022	0.167
22	Hahn et al. 2022	0.288	48	Ladenbauer et al. 2021	0.203	74	Solano et al. 2022	0.151
23	Hahn et al. 2022	0.277	49	Ladenbauer et al. 2021	0.469	75	Solano et al. 2022	0.180
24	Hahn et al. 2022	0.391	50	Ladenbauer et al. 2021	0.388	76	Solano et al. 2022	0.165
25	Hahn et al. 2022	0.286	51	Ladenbauer et al. 2021	0.229	77	Weiner et al. 2023	0.358
26	Hahn et al. 2022	0.378	52	Ladenbauer et al. 2021	0.3	78	Weiner et al. 2023	0.389

Figure 2: Pareto *k* diagnostic statistics for the spindle amplitude model

Pareto k diagnosis statistics for the strength model								
ESID	Study	Pareto k	ESID	Study	Pareto k	ESID	Study	Pareto k
1	Bastian et al. 2022	0.315	30	Hahn et al. 2020	0.485	59	Kurz et al. 2023	0.313
2	Bastian et al. 2022	0.434	31	Hahn et al. 2020	0.425	60	Ladenbauer et al. 2021	0.284
3	Bastian et al. 2022	0.401	32	Hahn et al. 2020	0.48	61	Ladenbauer et al. 2021	0.260
4	Bastian et al. 2022	0.404	33	Hahn et al. 2020	0.464	62	Ladenbauer et al. 2021	0.278
5	Cox et al. 2018	0.345	34	Hahn et al. 2022	0.271	63	Ladenbauer et al. 2021	0.278
6	Cox et al. 2018	0.459	35	Hahn et al. 2022	0.348	64	Mikutta et al. 2019	0.321
7	Cox et al. 2018	0.394	36	Hahn et al. 2022	0.281	65	Mikutta et al. 2019	0.305
8	Cox et al. 2018	0.332	37	Hahn et al. 2022	0.318	66	Mylonas et al. 2020	0.403
9	Cox et al. 2018	0.271	38	Hahn et al. 2022	0.475	67	Mylonas et al. 2022	0.319
10	Cox et al. 2018	0.363	39	Hahn et al. 2022	0.473	68	Niknazar et al. 2015	0.410
11	Cox et al. 2018	0.221	40	Halonen et al. 2021	0.349	69	Schreiner et al. 2021	0.394
12	Cox et al. 2018	0.396	41	Halonen et al. 2021	0.334	70	Solano et al. 2022	0.267
13	Cox et al. 2018	0.436	42	Halonen et al. 2022	0.703 *	71	Solano et al. 2022	0.228
14	Cox et al. 2018	0.425	43	Halonen et al. 2022	0.567	72	Solano et al. 2022	0.261
15	Cox et al. 2018	0.319	44	Halonen et al. 2022	0.659	73	Solano et al. 2022	0.191
16	Cox et al. 2018	0.253	45	Halonen et al. 2022	0.594	74	Solano et al. 2022	0.187
17	Denis et al. 2022	0.324	46	Helfrich et al. 2018	0.441	75	Solano et al. 2022	0.300
18	Denis et al. 2022	0.394	47	Helfrich et al. 2018	0.363	76	Solano et al. 2022	0.197
19	Denis et al. 2021	0.392	48	Kurz et al. 2021	0.317	77	Solano et al. 2022	0.187
20	Denis et al. 2021	0.441	49	Kurz et al. 2021	0.345	78	Solano et al. 2022	0.222
21	Denis et al. 2021	0.376	50	Kurz et al. 2021	0.288	79	Solano et al. 2022	0.300
22	Denis et al. 2021	0.324	51	Kurz et al. 2021	0.427	80	Solano et al. 2022	0.209
23	Denis et al. 2021	0.432	52	Kurz et al. 2021	0.42	81	Solano et al. 2022	0.285
24	Denis et al. 2021	0.407	53	Kurz et al. 2021	0.276	82	Weiner et al. 2023	0.362
25	Donnelly et al. 2022	0.248	54	Kurz et al. 2023	0.413	83	Weiner et al. 2023	0.414
26	Donnelly et al. 2022	0.353	55	Kurz et al. 2023	0.464	84	Zhang et al. 2020	0.404
27	Donnelly et al. 2022	0.197	56	Kurz et al. 2023	0.412	85	Zhang et al. 2020	0.380
28	Hahn et al. 2020	0.477	57	Kurz et al. 2023	0.347	86	Zhang et al. 2020	0.404
29	Hahn et al. 2020	0.496	58	Kurz et al. 2023	0.376	NA	NA	NA

Figure 3: Pareto *k* diagnostic statistics for the coupling strength model

Pareto k diagnosis statistics for the percentage model								
ESID	Study	Pareto k	ESID	Study	Pareto k	ESID	Study	Pareto k
1	Bastian et al. 2022	0.228	16	Hahn et al. 2020	0.471	31	Schreiner et al. 2021	0.488
2	Bastian et al. 2022	0.251	17	Halonen et al. 2021	0.479	32	Solano et al. 2022	0.304
3	Bastian et al. 2022	0.289	18	Halonen et al. 2021	0.437	33	Solano et al. 2022	0.308
4	Bastian et al. 2022	0.385	19	Halonen et al. 2022	0.814 *	34	Solano et al. 2022	0.159
5	Denis et al. 2022	0.466	20	Halonen et al. 2022	0.702 *	35	Solano et al. 2022	0.221
6	Denis et al. 2022	0.524	21	Halonen et al. 2022	0.543	36	Solano et al. 2022	0.314
7	Denis et al. 2021	0.406	22	Halonen et al. 2022	0.754 *	37	Solano et al. 2022	0.255
8	Denis et al. 2021	0.461	23	Kurz et al. 2021	0.355	38	Solano et al. 2022	0.284
9	Denis et al. 2021	0.553	24	Kurz et al. 2021	0.347	39	Solano et al. 2022	0.332
10	Denis et al. 2021	0.466	25	Kurz et al. 2021	0.331	40	Solano et al. 2022	0.237
11	Denis et al. 2021	0.296	26	Kurz et al. 2021	0.309	41	Solano et al. 2022	0.167
12	Denis et al. 2021	0.361	27	Kurz et al. 2021	0.374	42	Solano et al. 2022	0.319
13	Hahn et al. 2020	0.295	28	Kurz et al. 2021	0.322	43	Solano et al. 2022	0.252
14	Hahn et al. 2020	0.403	29	Mylonas et al. 2020	0.462	NA	NA	NA
15	Hahn et al. 2020	0.540	30	Mylonas et al. 2022	0.493	NA	NA	NA

Figure 4: Pareto *k* diagnostic statistics for the coupling percentage model

Notes. Pareto *k* can be interpreted in a similar way to Cook's distance in frequentist analysis. When an effect size has a Pareto *k* value larger than 0.7 or even 1.0, it can be classified as an influential data point and might introduce the issue of misspecification of the model. Consequently, we excluded all studies with Pareto *k* values larger than 0.7 from the focal analysis. ESID corresponds to the esid column labeled in Source data of Figure 4, 6-8.
