# Supplementary material for: Bayesian meta-analysis reveals the mechanistic role of slow oscillation-spindle coupling in sleep-dependent memory consolidation
Source: eLife. 2025 Oct 8;13:RP101992. doi: 10.7554/eLife.101992 (PMC12507438; doi:10.7554/eLife.101992)
Supplement: Supplementary file 5. — Forest plots generated using frequentist analysis for each fitted overall Bayesian model. [file elife-101992-supp5.pdf]

Supplementary File 5: Frequentist analysis

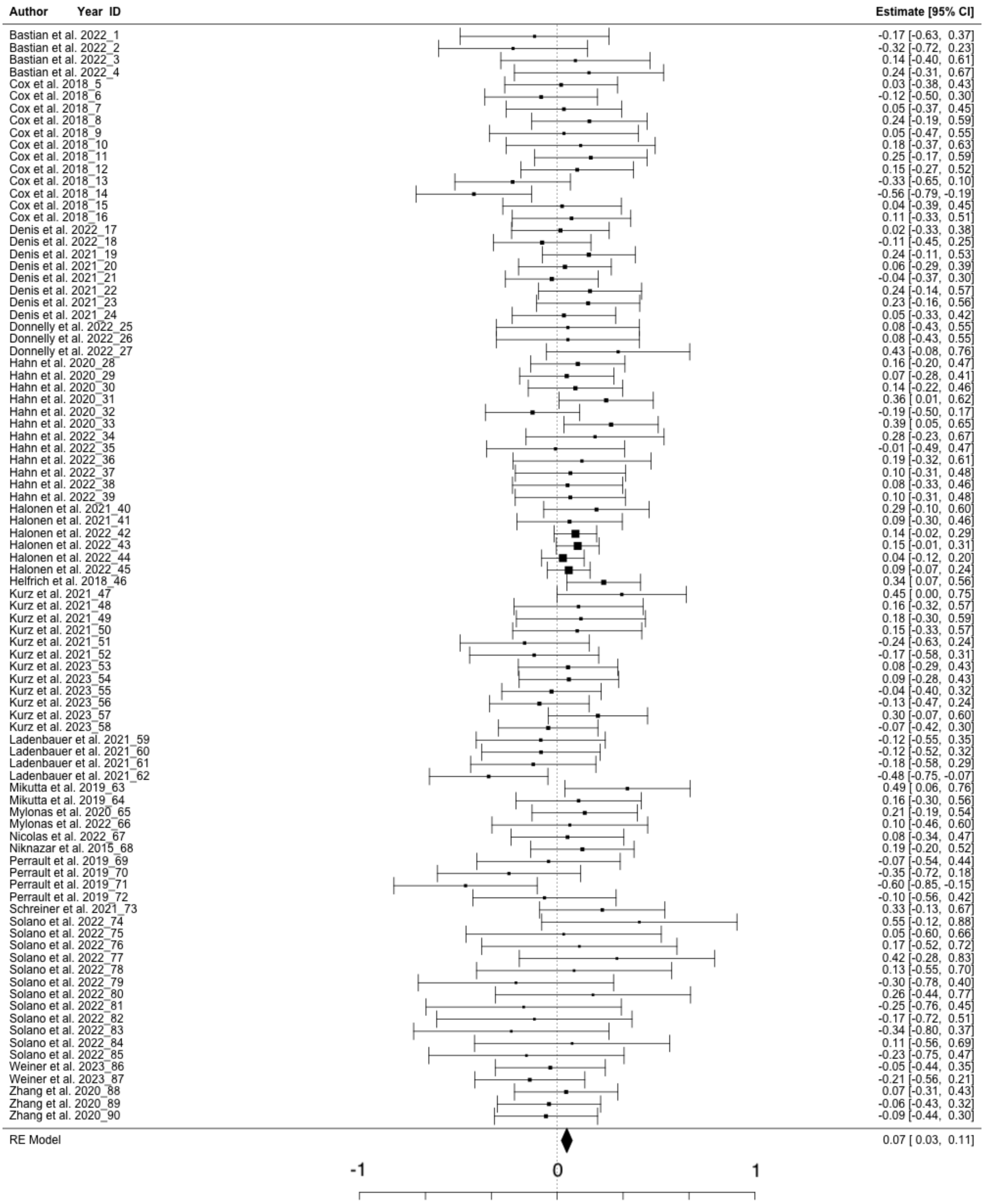

Figure 1: Coupling phase frequentist forest plot

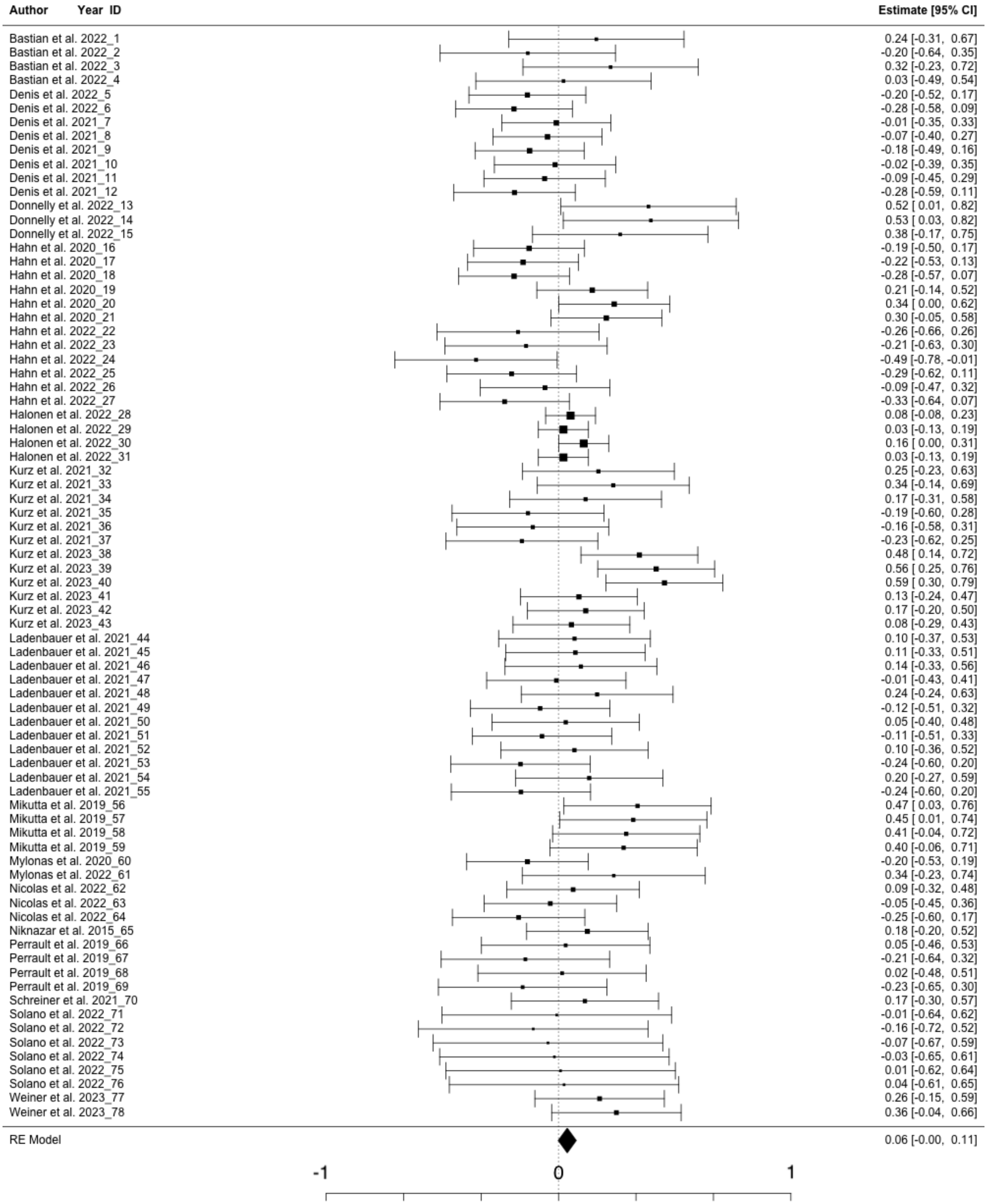

Figure 2: SP amplitude frequentist forest plot

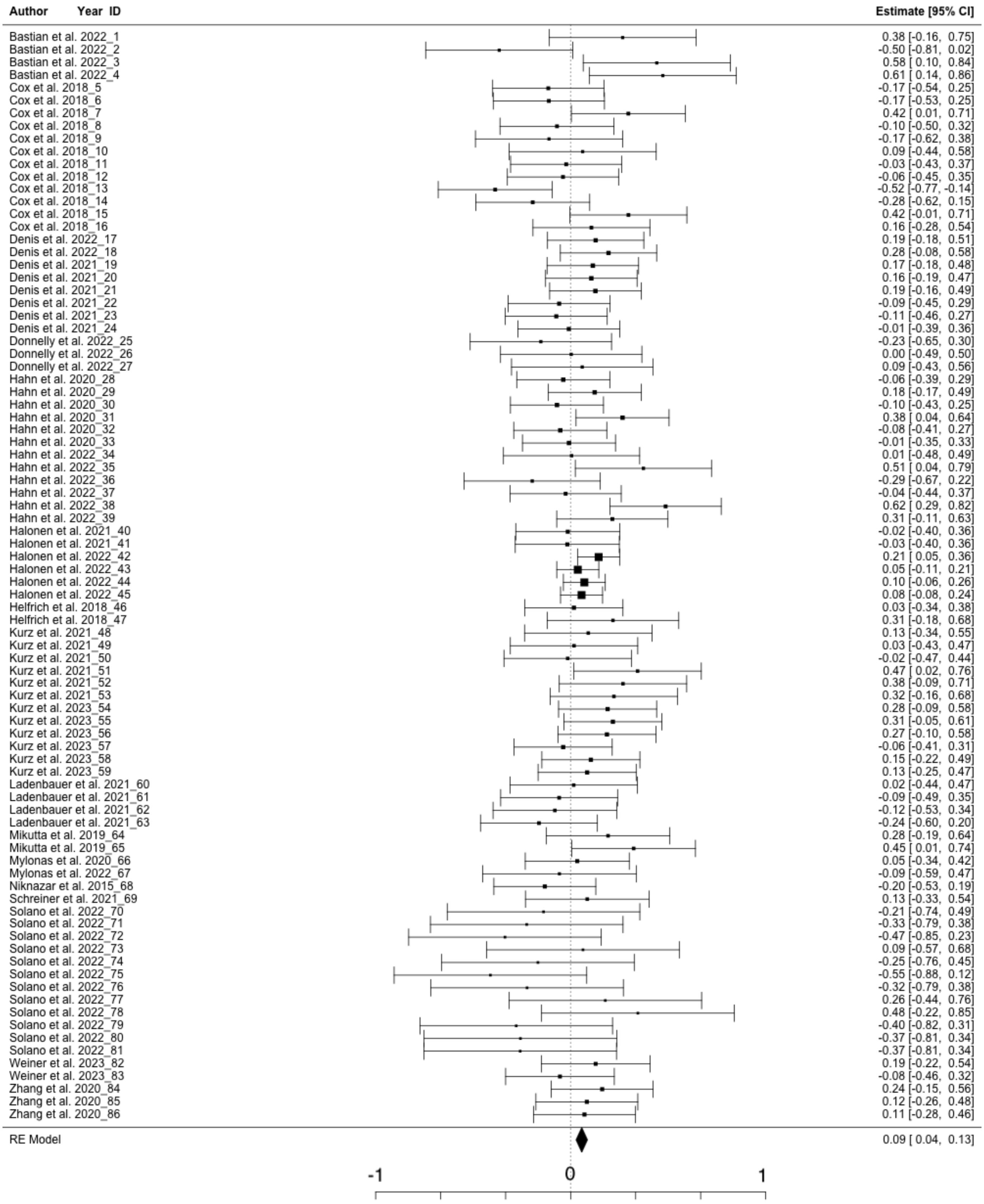

Figure 3: Coupling strength frequentist forest plot

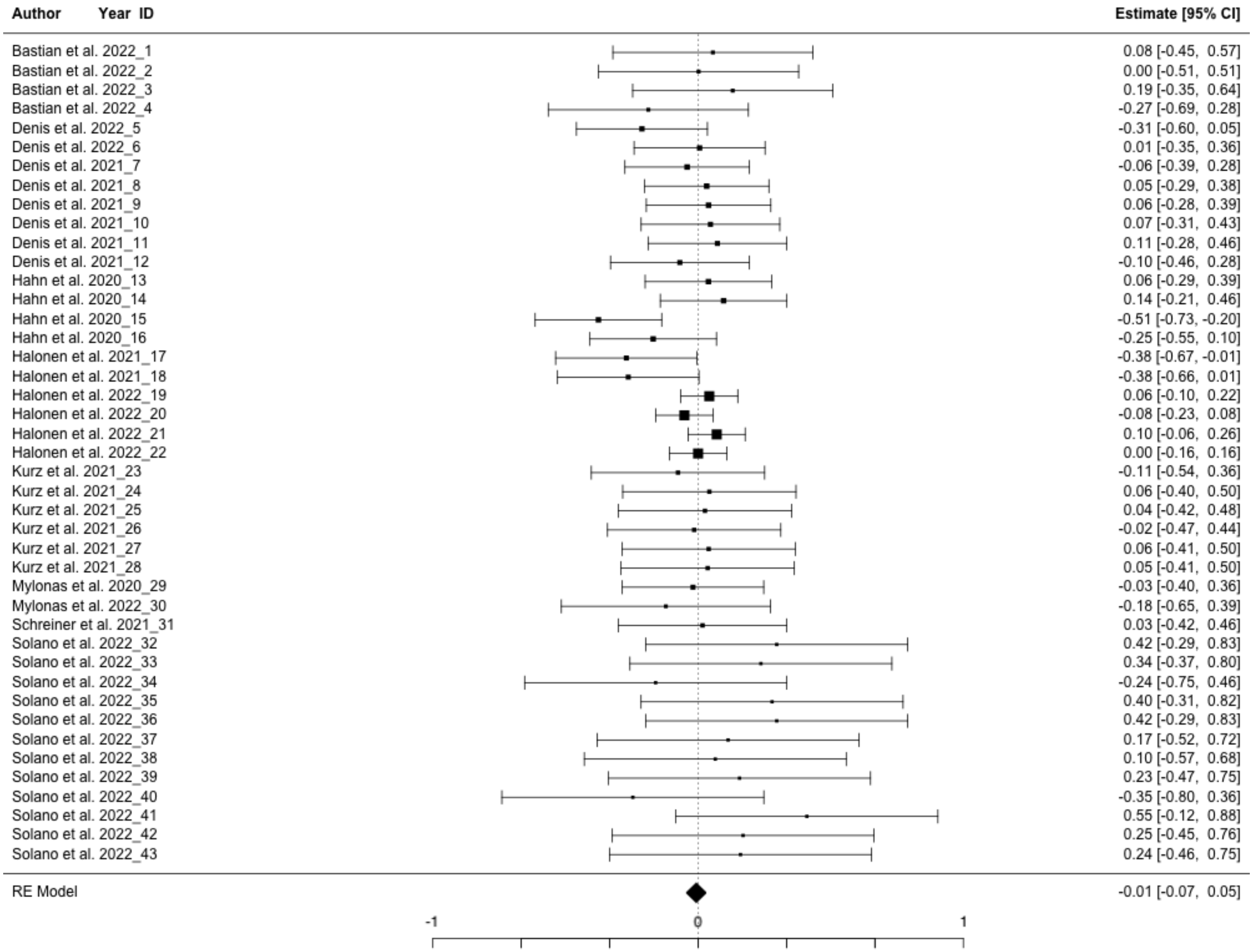

Figure 4: Coupling percentage frequentist forest plot
