# Supplementary material for: Bayesian meta-analysis reveals the mechanistic role of slow oscillation-spindle coupling in sleep-dependent memory consolidation
Source: eLife. 2025 Oct 8;13:RP101992. doi: 10.7554/eLife.101992 (PMC12507438; doi:10.7554/eLife.101992)
Supplement: Supplementary file 6. [file elife-101992-supp6.pdf]

# Supplementary File 6: Posterior distribution of moderators

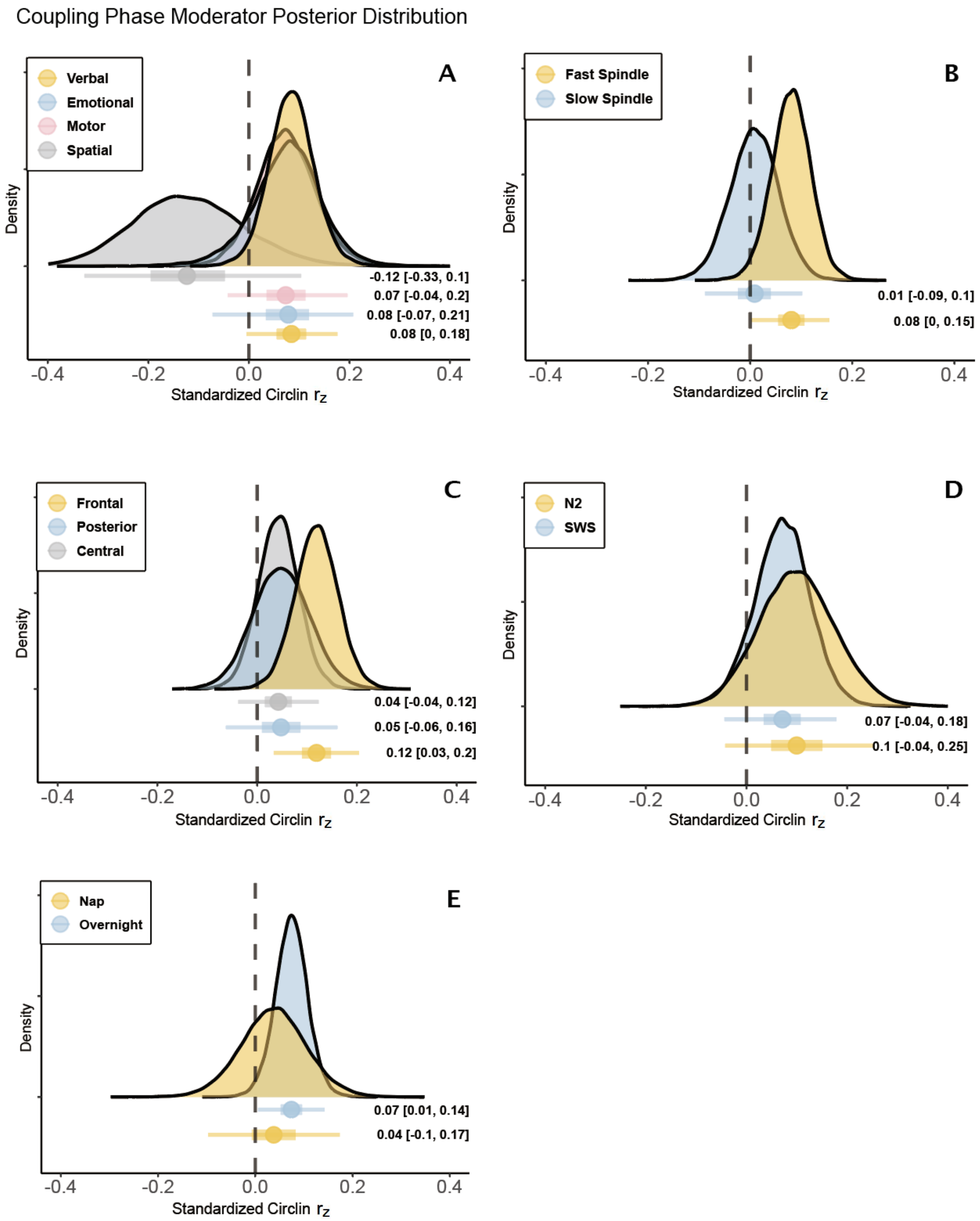


Figure 1: Posterior distributions of moderation factors of the coupling phase

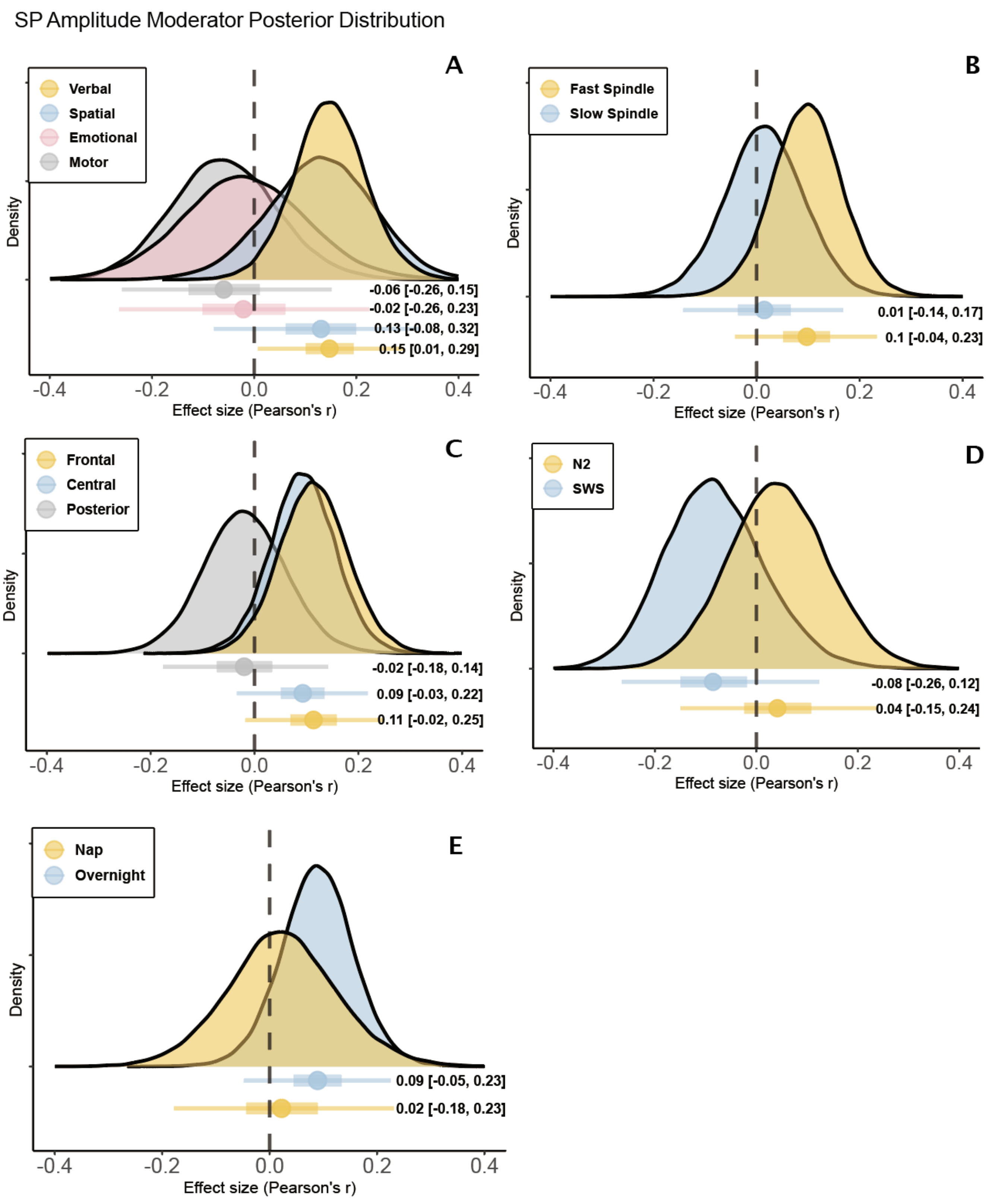


Figure 2: Posterior distributions of moderation factors of the SP amplitude

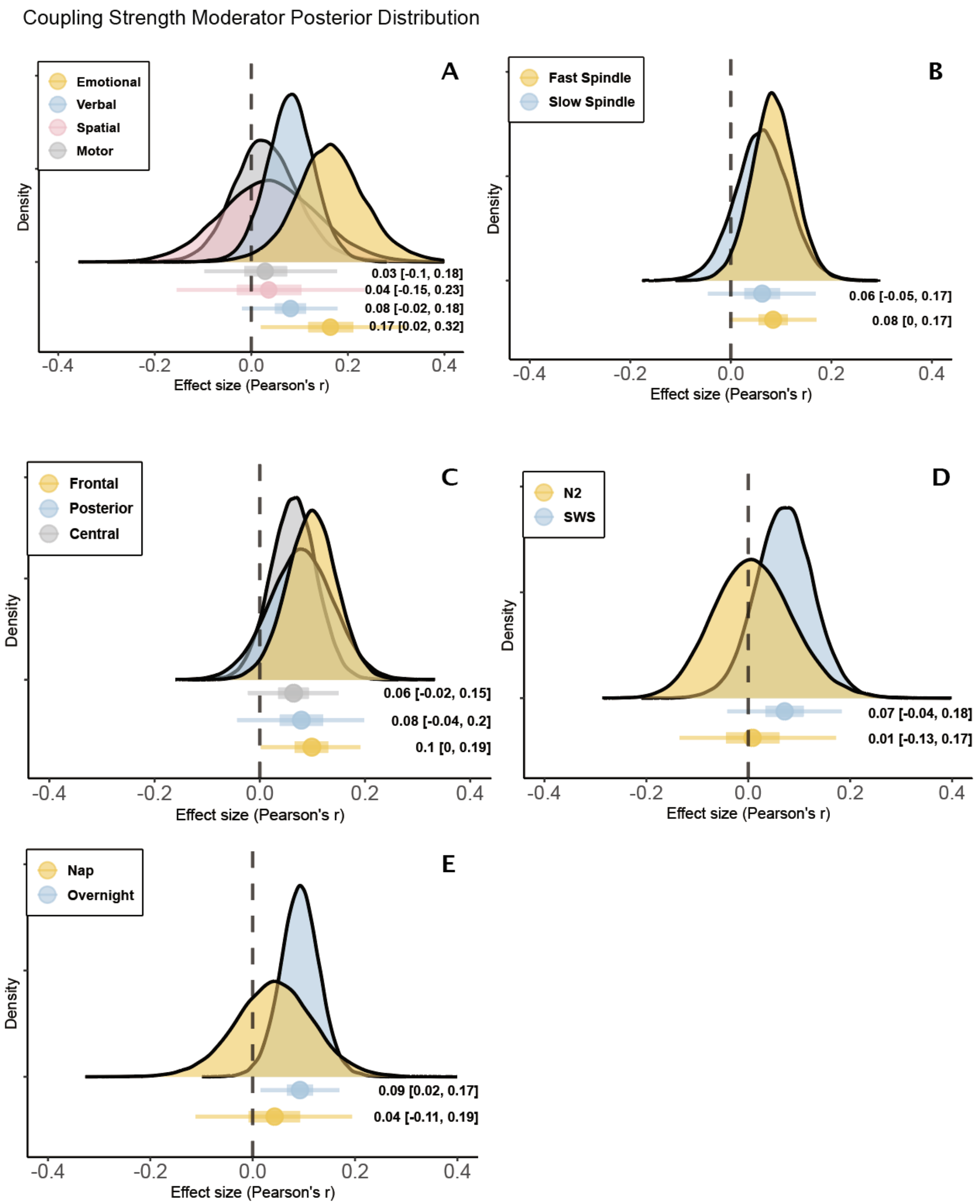


Figure 3: Posterior distributions of moderation factors of the coupling strength

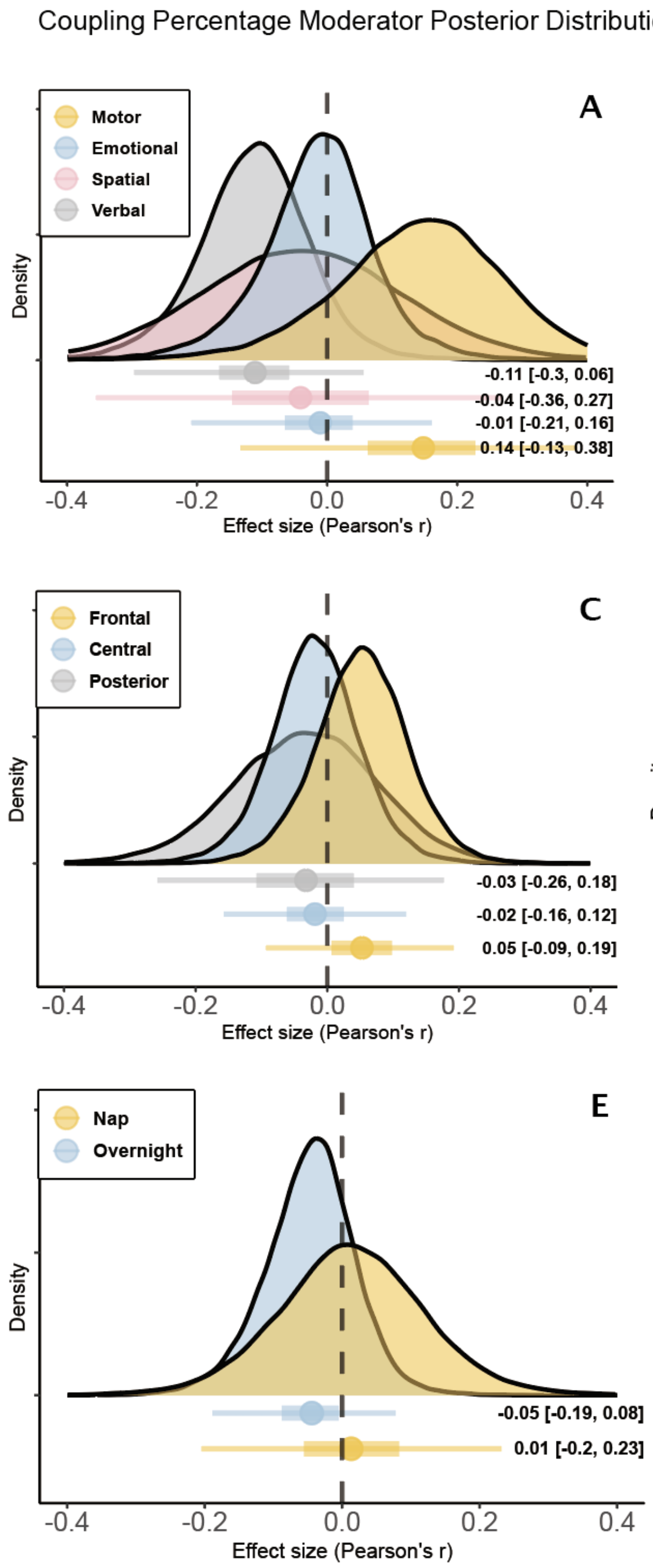


Figure 4: Posterior distributions of moderation factors of the coupling percentage

Notes. The vertical line reflects the mean correlation coefficient under the null hypothesis. Colored dots and error bars reflect the mean and 95% credible intervals of each posterior distribution.
